# Effect of arsenic on the risk of gestational diabetes mellitus: a systematic review and meta-analysis

**DOI:** 10.1186/s12889-024-18596-6

**Published:** 2024-04-23

**Authors:** Rui Wu, Min Duan, Dongsheng Zong, Zuojing Li

**Affiliations:** 1 School of Life Sciences and Biopharmaceuticals, Shenyang Pharmaceutical University, Shenyang, China; 2 School of Medical Devices, Shenyang Pharmaceutical University, Shenyang, China

**Keywords:** Arsenic, Pregnancy, GDM, Gestational diabetes, Meta-analysis

## Abstract

**Background:**

Gestational diabetes mellitus (GDM) is a complication of pregnancy associated with numerous adverse outcomes. There may be a potential link between GDM and arsenic (As) exposure, but this hypothesis remains controversial. This meta-analysis summarizes the latest studies evaluating the association between As and GDM.

**Methods:**

A comprehensive search of the PubMed, Embase, and Scopus databases up to September 2023 was performed. The pooled estimates with 95% CIs were presented using forest plots. Estimates were calculated with random effects models, and subgroup and sensitivity analyses were conducted to address heterogeneity.

**Results:**

A total of 13 eligible studies involving 2575 patients with GDM were included in this meta-analysis. The results showed that women exposed to As had a significantly increased risk of GDM (OR 1.47, 95% CI: 1.11 to 1.95, *P* = 0.007). Subgroup analyses suggested that the heterogeneity might be attributed to the years of publication. In addition, sensitivity analysis confirmed the robust and reliable results.

**Conclusions:**

This analysis suggested that women exposed to As have a greater risk of GDM. However, the significant heterogeneity across studies requires careful interpretation.

**Registration:**

The PROSPERO registration ID is CRD42023461820.

**Supplementary Information:**

The online version contains supplementary material available at 10.1186/s12889-024-18596-6.

## Introduction

Gestational diabetes mellitus (GDM) is a metabolic disorder that occurs during pregnancy and affects approximately 15% of all pregnancies [1]. This disease usually appears in the late second and early third trimesters [2, 3]. Medical professionals strive to reduce the incidence of GDM by employing strategies focused on effective screening, timely diagnosis, and careful management [4]. These methods include monitoring glycemic levels, balanced nutrition, regular exercise, and medication support [5–8]. The primary goal of these interventions is to significantly reduce the recurrence of GDM in subsequent pregnancies. In addition, enabling women to master the necessary knowledge of managing glycemic levels can also help reduce the recurrence of GDM.

Many factors contribute to GDM, including genetic predispositions, environmental influences, and other possible causes [9]. The potential link between exposure to environmental pollutants and GDM has been the subject of many studies, but this link remains controversial [10]. To reduce the incidence of GDM, raising awareness of the potential risks of environmental pollutants during pregnancy is essential. Developing relevant strategies, such as improving air quality and reducing exposure to hazardous chemicals, could reduce the risk of GDM in future pregnancies.

Arsenic (As) is a nonbiodegradable heavy metal that can accumulate in the human body and lead to toxicity [11–13]. The main sources of As include fruits, vegetables, grains, seafood, groundwater, industrial emissions, and waste materials [14–16]. Previous studies have hypothesized that As might increase the risk of GDM [17–21]. However, recent studies have shown that As does not significantly increase the risk of GDM [22–25]. Thus, the current conclusion is still controversial. Therefore, this meta-analysis aimed to summarize different perspectives and provide an updated overview of the available evidence to investigate the relationship between As exposure and the risk of GDM.

## Methods

This meta-analysis was reported following the Preferred Reporting Items for Systematic Reviews and Meta-Analysis (PRISMA) [26] and registered in PROSPERO (Registration ID: CRD42023461820).

### Literature search

A comprehensive search for studies was conducted in the PubMed, Embase, and Scopus databases from inception through September 8, 2023. The search was limited to studies published in English. The search strategy included the keywords “Diabetes, Gestational”, “Diabetes, Pregnancy-Induced”, “Gestational Diabetes”, “Diabetes Mellitus, Gestational”, “Gestational Diabetes Mellitus”, “Arsenic”, and “Arsenic-75”. The MeSH terms and Boolean operators were used to develop a robust search strategy. The detailed search strategies for each database are provided in Supplementary 1. Studies identified through a systematic search were retrieved and managed using EndNote software version X9 (Clarivate Analytics, Philadelphia, USA, 2013). All literature searches were conducted by two independent reviewers.

### Inclusion criteria

Studies were included if they met the following criteria: (1) studies that evaluated As exposure through appropriate exposure indicators (blood, urine, hair, tap water, and meconium); (2) studies used odds ratios (ORs) and 95% confidence intervals (CIs) for risk estimation; (3) studies were designed as observational studies (cross-sectional, case-control, cohort, retrospective case-control studies nested in a cohort, or correlations); (4) studies reported data on GDM in humans and focused on adults; and (5) studies that diagnosis of GDM according to the criteria of the American Diabetes Association (ADA), World Health Organization (WHO), Collège National des Gynécologues et Obstétriciens Français (CNGOF), Canadian Diabetes Association and Society of Obstetricians and Gynecologists of Canada (CDA-SOGC), or the Ministry of Health (MOH) of China.

### Exclusion criteria

Studies were excluded if they met the following criteria: (1) studies were reviews, meta-analyses, case reports,

or randomized controlled trials; (2) non-human studies; (3) data in the studies were incomplete, insufficient, or reused; and (4) duplicate studies or full articles unavailable.

### Risk of bias assessment

Two reviewers carefully assessed the risk of bias in the studies. For cohort and case-control studies, the risk of bias was evaluated using the Newcastle-Ottawa Scale (NOS) [27], which has a total score of 9 points; studies scoring 0–4 were defined as low quality, 5–6 as moderate quality, and 7–9 as high quality. Cross-sectional studies were assessed through the Agency for Healthcare Research and Quality (AHRQ) criteria [28], which has a total score of 11 points; studies scoring 0–3 were defined as low quality, 4–7 as moderate quality, and 8–11 as high quality.

### Data extraction

Two independent reviewers extracted relevant data for each study, and any discrepancies between reviewers were resolved through discussion. The following information was extracted for the included studies: the first author, publication year, country, type of study, diagnostic criteria, type of sample, number of participants, limit of detection (LOD), and cutoff number.

### Statistical analysis

Dichotomous data were expressed as ORs and 95% CIs; for studies disclosing binary, tertile, or quartile risk evaluations, only the most elevated data were incorporated. Risk evaluations were converted to log ORs and analyzed using fixed or random-effects models. Differences were considered to be statistically significant when *P* < 0.05.

Statistical heterogeneity was measured using the chi-squared test and *I*^2^ statistic. A *P* value less than 0.10 indicated the presence of heterogeneity, and the heterogeneity was classified as low, moderate, or high if *I*^2^ was < 50%, 50–75%, or > 75%, respectively. Considering the expected heterogeneity, the estimates were calculated with DerSimonian–Laird’s random effect models [29]. All the statistical calculations were performed using Review Manager version 5.4 (Cochrane Collaboration, Software Update, Oxford, UK). Publication bias was evaluated using funnel plots and Egger’s test in Stata software 12.0 (StataCorp, College Station, TX, USA), and the analysis was restricted to studies with a sample size of 10 or more.

Subgroup analyses were performed to explore the impact of country, study type, diagnostic criteria, test samples, cutoff points, median year of publication, and quality on heterogeneity. Analyses were conducted only when the subgroup included a minimum of two studies. In addition, sensitivity analysis was conducted to evaluate each study’s overall impact and test the reliability of the results.

## Results

### Study selection and characteristics

**Fig. 1:**
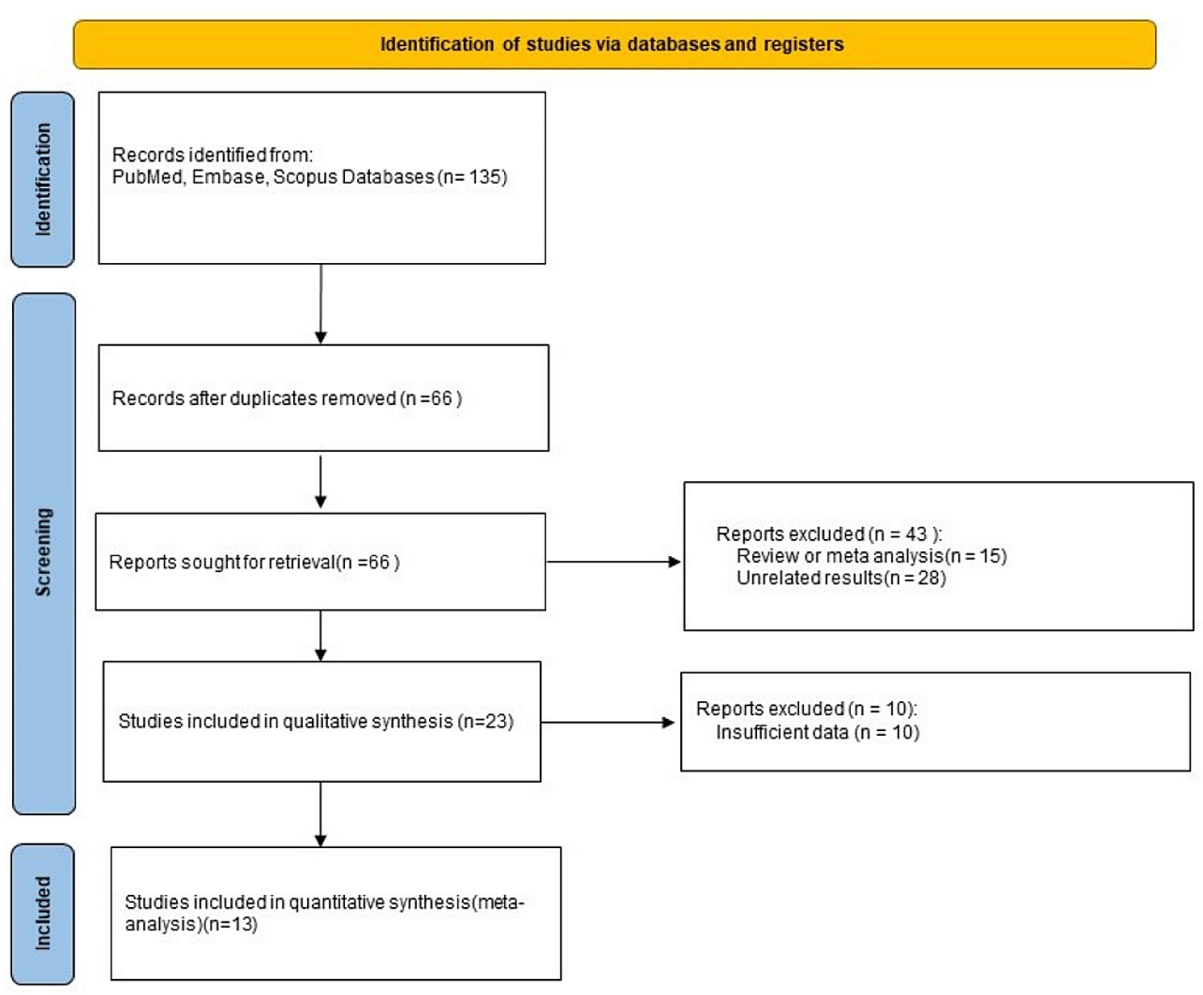
Included in the flow chart

From the comprehensive database search, 135 articles were initially identified, and after the exclusion of duplicate studies, 66 remained for further consideration. Next, 43 articles were excluded for review or meta-analysis. After a thorough review of the full texts of the remaining 23 articles, it was found that ten did not have sufficient data, leading to the final inclusion of 13 articles [17–25, 30–33]. The search and selection process is depicted in Fig. 1, and the basic characteristics of the included studies are detailed in Table 1.

The quantitative analyses included 16,792 participants, 2,575 in the case group and 14,217 in the control group. Thirteen included studies were conducted across six countries: China, Canada, the USA, Bangladesh, Chile, and France.

### Outcomes

**Fig. 2:**
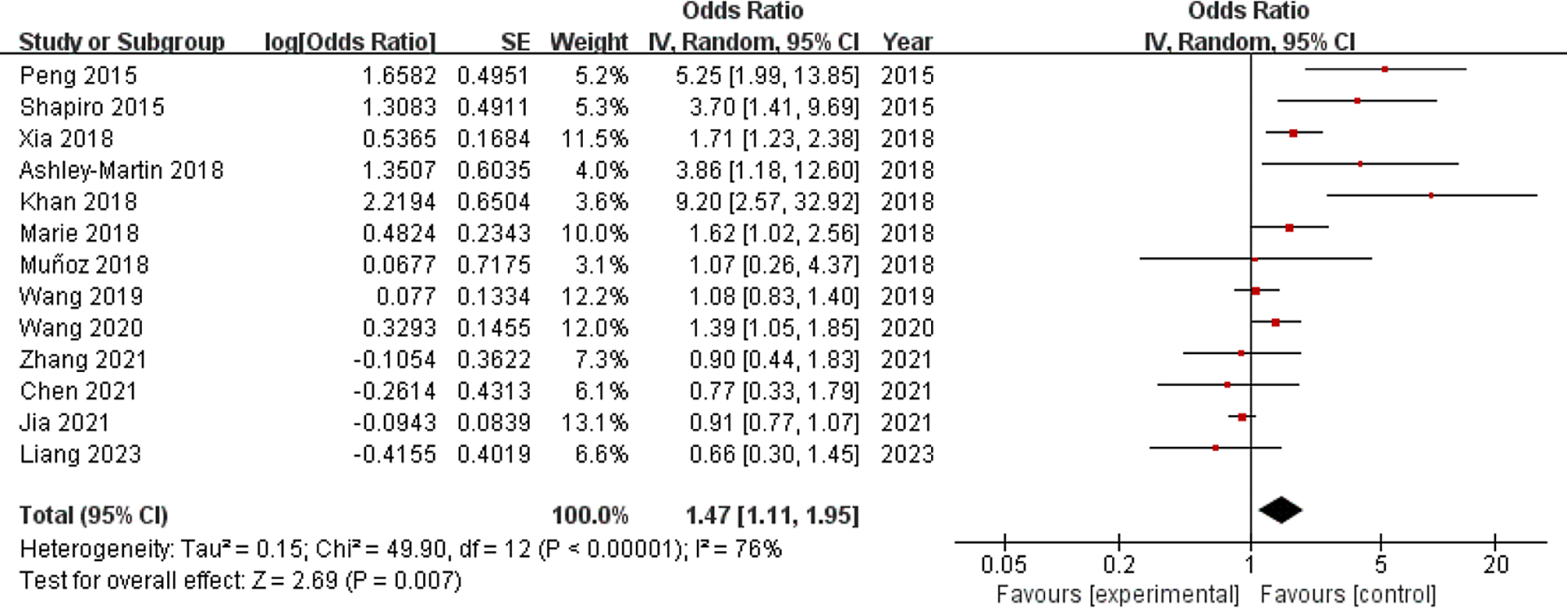
Forest plot of all studies included in the quantitative synthesis

**Fig. 3:**
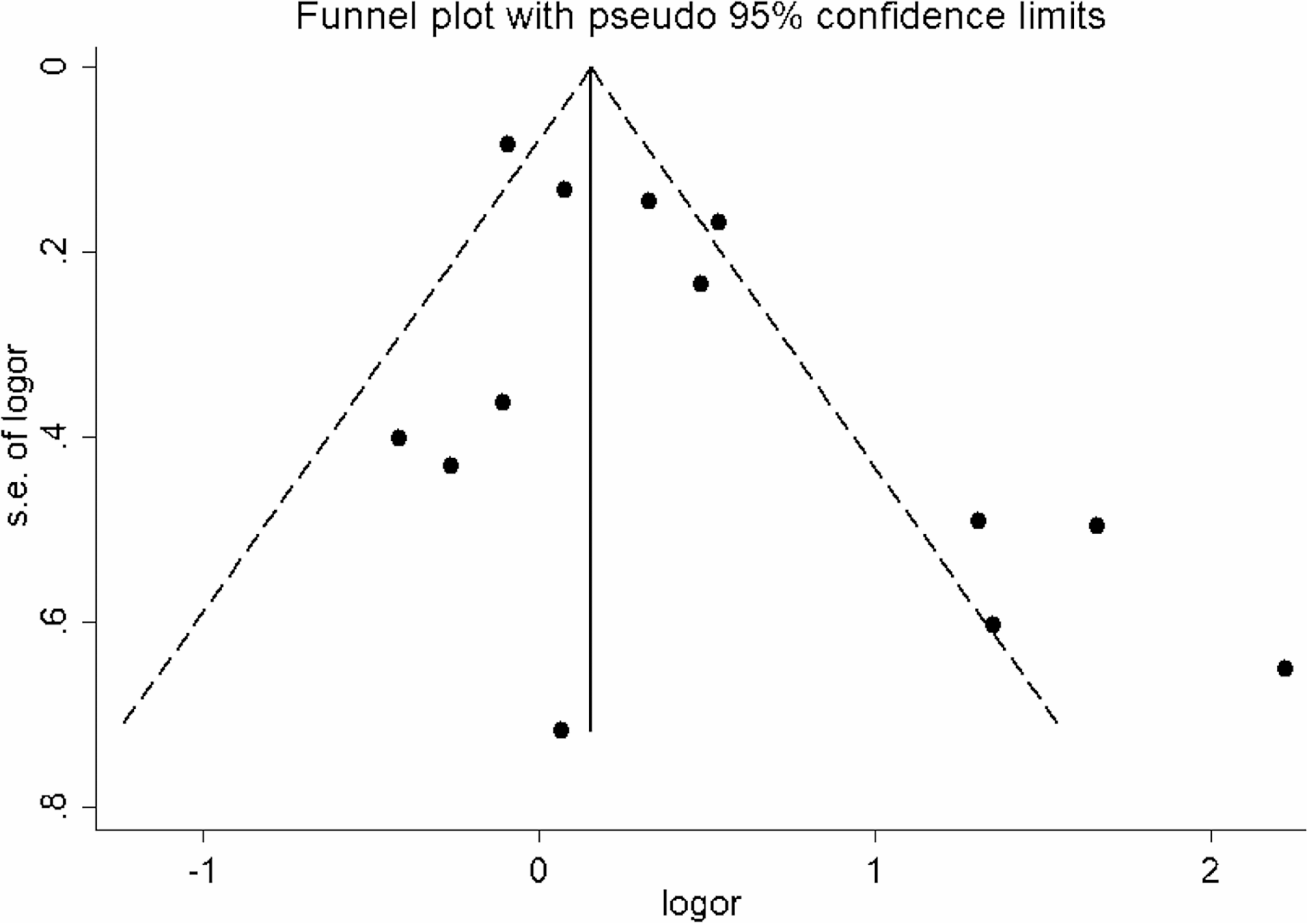
Funnel plot of all studies included in the quantitative synthesis

The forest plot of the meta-analysis included 13 studies (Fig. 2). The results showed that exposure to As significantly increased the risk of GDM in women (OR 1.47, 95% CI: 1.11 to 1.95, *P* = 0.007). Nonetheless, the significant heterogeneity (*I*^2^ = 76%) requires careful explanation of these findings. Moreover, the funnel plot (Fig. 3) combined with Egger’s test (t = 2.33, *P* = 0.04) indicated publication bias in this analysis.

**Table 1:** Basic characteristics of the eligible studies

Study	Year	Country	Study type	Diagnostic criteria	Test samples	Case group (N)	Control group (N)	Gestational week	LOD(µg/L)	Cutoffs
Peng	2015	China	Retrospective case-control study nested in a cohort	WHO Criteria [34]	Meconium	137	190	24–28	0.06 µg/L	quartile
Shapiro	2015	Canada	Cohort study	CDA-SOGC Criteria [35, 36]	Blood	48	1167	first trimester	0.22 µg/L	quartile
Ashley-Martin	2018	Canada	Cohort study	CDA-SOGC Criteria [35, 36]	Urine	42	1049	first trimester	0.75 µg/L	tertile
Khan	2018	Bangladesh	Cross-sectional study	WHO Criteria [34]	Urine	31	169	29.44 ± 3.20	Not As exposed: ≤ 0.100 mg/L As exposed: > 0.100 mg/L	not said
Marie	2018	French	Semiecological study	CNGOF Criteria [37]	Tap water	286	4767	24–28	Not As exposed: < 10 µg/L As Exposed: a. Low 10–30 µg/L b. High ≥ 30 µg/L	not said
Muñoz	2018	Chile	Cross-sectional study	WHO Criteria [34]	Urine	21	223	24–28	0.1 µg/L	tertile
Xia	2018	China	Cohort study	ADA Criteria [38]	Blood	419	2841	39.03 ± 1.39	0.0047 µg/L	quartile
Wang	2019	China	Cohort study	ADA Criteria [38]	Blood	776	776	≥ 29	As level: a. Low < 10.64 µg/L b. Middle 10.64–21.12 µg/L c. High ≥ 21.12 µg/L	tertile
Wang	2020	China	Cohort study	ADA Criteria [38]	Urine	241	1849	< 20	0.009 µg/L CAU-As: a. Low < 32.11 µg/L b. Middle 32.11–48.11 µg/L c. High ≥ 48.11 µg/L	tertile
Chen	2021	America	Case-control study	ADA Criteria [38]	Urine	64	237	24–28	1.25 µg/L	tertile
Jia	2021	China	Retrospective case-control study nested in a cohort	ADA Criteria [38]	Hair	335	343	< 20	0.011 µg/L	binary
Zhang	2021	China	Cross-sectional study	MOH Criteria [39]	Urine	89	307	24–28	0.2 µg/L for the As^3+^, MMA, DMA, and AsB, and 0.5 µg/L for As^5+^.	quartile
Liang	2023	China	Cross-sectional study	MOH Criteria [39]	Urine	86	299	24–28	As^3+^, MMA, DMA, and AsB were 0.2 µg/L and 0.5 µg/L for As^5+^	tertile

*Abbreviations*: As, arsenic; LOD, limit of detection; WHO, World Health Organization; CDA-SOGC, Canadian Diabetes Association-Society of Obstetricians and Gynecologists of Canada; CNGOF, French National College of Obstetricians and Gynecologists; ADA, American Diabetes Association; MOH, Ministry of Health of China; CAU-As, creatinine-adjusted urinary arsenic; MMA, monomethylarsonic acid; DMA, dimethylarsinic acid; AsB, and arsenobetaine

### Sensitivity analysis

Sensitivity analysis was conducted by omitting one at a time, which further indicated that the pooled results were.

stable (Table 2). However, heterogeneity remained significant, with *I*^2^ values fluctuating between 68% and 78%.

**Table 2:** Sensitivity analysis

Study	Number (N)	OR (95% CI)	Heterogeneity (*I*^2^%)	*P*
All studies	13	1.47 (1.11, 1.95)	76	0.007
All studies exclude Jia 2021	12	1.58 (1.17, 2.15)	68	0.003
All studies exclude Marie 2018	12	1.46 (1.08, 1.98)	77	0.01
All studies exclude Khan 2018	12	1.36 (1.05, 1.77)	72	0.02
All studies exclude Xia 2018	12	1.45 (1.07, 1.96)	75	0.02
All studies exclude Shapiro 2015	12	1.39 (1.05, 1.84)	75	0.02
All studies exclude Peng 2015	12	1.36 (1.04, 1.77)	73	0.03
All studies exclude Zhang 2021	12	1.54 (1.14, 2.07)	78	0.005
All studies exclude Wang 2019	12	1.57 (1.13, 2.18)	78	0.008
All studies exclude Chen 2021	12	1.54 (1.15, 2.06)	78	0.004
All studies exclude Wang 2020	12	1.51 (1.09, 2.08)	77	0.01
All studies exclude Ashley-Martin 2018	12	1.41 (1.06, 1.86)	76	0.02
All studies exclude Liang 2023	12	1.56 (1.16, 2.09)	77	0.003
All studies exclude Muñoz 2018	12	1.49 (1.12, 1.99)	78	0.007

### Subgroup analysis

Subgroup analyses were mainly based on country, study type, diagnostic criteria, sample type, cutoff values, median year of publication, and study quality (Table 3).

By pooling data from multiple countries, we found notable differences. Studies from China showed significant heterogeneity (*I*^2^ = 77%, *P* = 0.16), while studies from Canada showed no heterogeneity (*I*^2^ = 0, *P* = 0.0005). The comprehensive risk estimation of studies from Canada showed statistical significance (OR 3.76, 95% CI: 1.78 to 7.94), while studies from China did not show statistical significance (OR 1.23, 95% CI: 0.92 to 1.65).

Subgrouping by study type revealed varying levels of heterogeneity. Retrospective case-control studies displayed the highest heterogeneity (*I*^2^ = 92%, *P* = 0.41), followed by cross-sectional studies (*I*^2^ = 76%, *P* = 0.49) and cohort studies (*I*^2^ = 66%, *P* = 0.006). Among these, only the results from cohort studies were statistically significant (OR 1.59, 95% CI: 1.14 to 2.22).

When analyzing the diagnostic criteria for GDM, the studies using the CDA-SOGC (*I*^2^ = 0, *P* = 0.0005) and MOH criteria (*I*^2^ = 0, *P* = 0.36) showed no heterogeneity. In contrast, studies following the ADA (*I*^2^ = 74%, *P* = 0.24) and WHO criteria (*I*^2^ = 62%, *P* = 0.02) revealed significant heterogeneity. Only studies employing the CDA-SOGC (OR 3.76, 95% CI: 1.78 to 7.94) and WHO criteria (OR 3.93, 95% CI: 1.27 to 12.22) demonstrated statistical significance. Conversely, studies based on the ADA (OR 1.17, 95% CI: 0.90 to 1.52) and MOH criteria (OR 0.78, 95% CI: 0.46 to 1.33) did not reveal statistical significance.

The test samples showed notable heterogeneity in blood (*I*^2^ = 78%, *P* = 0.07) and urine samples (*I*^2^ = 67%, *P* = 0.22). Moreover, the analysis revealed that none of the pooled effects reached statistical significance (OR 1.61, 95% CI: 0.97 to 2.68 for blood samples; OR 1.39, 95% CI: 0.82 to 2.34 for urine samples).

An examination revealed that only the cutoffs grouped into quartiles were significantly different (OR 2.13, 95% CI: 1.11 to 4.10). However, considerable heterogeneity remained (*I*^2^ = 72%, *P* = 0.02). In contrast, studies with tertiles (OR 1.61, 95% CI: 0.85 to 1.56) or unreported cutoff values (OR 3.47, 95% CI: 0.64 to 18.81) did not demonstrate statistical significance. The heterogeneity in studies with triplets (*I*^2^ = 42%, *P* = 0.35) was approximately half that seen in studies with unreported cutoff values (*I*^2^ = 84%, *P* = 0.15).

The heterogeneity of both subgroups decreased during the year of publication, indicating that the year of publication was a reasonable source of heterogeneity. Heterogeneity was measured for studies conducted before 2018 (*I*^2^ = 59%, *P* < 0.0001) and after 2018 (*I*^2^ = 39%, *P* = 0.82). However, statistical significance was observed only in the pooled results from studies conducted before 2018 (OR 2.61, 95% CI: 1.65 to 4.13), while those conducted after 2018 did not show statistical significance (OR 1.02, 95% CI: 0.85 to 1.24).

Furthermore, study quality was classified as moderate or high. Heterogeneity persisted (*I*^2^ = 77%, *P* = 0.002 vs. *I*^2^ = 71%, *P* = 0.56). The pooled results of moderate-quality studies were statistically significant (OR 2.51, 95% CI: 1.40 to 4.50); however, high-quality studies were not significantly different (OR 1.09, 95% CI: 0.81 to 1.48).

**Table 3:** Results of subgroup analyses based on country, study type, diagnostic criteria, test sample, and cutoff values

Subgroup	Number (N)	OR (95% CI)	Heterogeneity (*I*^2^%)	*P*
**Country**				
China	7	1.23 (0.92, 1.65)	77	0.16
Canada	2	3.76 (1.78, 7.94)	0	0.0005
America	1	/	/	/
Bangladesh	1	/	/	/
Chile	1	/	/	/
French	1	/	/	/
**Study Type**				
Cohort study	5	1.59 (1.14, 2.22)	66	0.006
Cross-sectional study	4	1.43 (0.52, 3.92)	76	0.49
Retrospective case-control study nested in a cohort	2	2.04 (0.37, 11.32)	92	0.41
Case-control study	1	/	/	/
Semi-ecological study	1	/	/	/
**Diagnostic Criteria**				
ADA Criteria	5	1.17 (0.90, 1.52)	74	0.24
CDA-SOGC Criteria	2	3.76 (1.78, 7.94)	0	0.0005
MOH Criteria	2	0.78 (0.46, 1.33)	0	0.36
WHO Criteria	3	3.93 (1.27, 12.22)	62	0.02
CNGOF Criteria	1	/	/	/
**Test Samples**				
Blood	3	1.61 (0.97, 2.68)	78	0.07
Urine	7	1.39 (0.82, 2.34)	67	0.22
Hair	1	/	/	/
Tap water	1	/	/	/
Meconium	1	/	/	/
**Cutoffs**				
Tertile	6	1.16 (0.85, 1.56)	42	0.35
Quartile	4	2.13 (1.11, 4.10)	72	0.02
Not said	2	3.47 (0.64, 18.81)	84	0.15
Binary	1	/	/	/
**Median year of publication**				
Before 2018	7	2.61 (1.65, 4.13)	59	0.0001
After 2018	6	1.02 (0.85, 1.24)	39	0.82
**Quality**				
Moderate	7	2.51 (1.40, 4.50)	77	0.002
High	6	1.09 (0.81, 1.48)	71	0.56

*Abbreviations*: WHO, World Health Organization; CDA-SOGC, Canadian Diabetes Association-Society of Obstetricians and Gynecologists of Canada; CNGOF, French National College of Obstetricians and Gynecologists; ADA, American Diabetes Association; MOH, Ministry of Health of China

### Assessment of study quality

Each study was evaluated using corresponding scales. A retrospective case-control study nested in a cohort characterized by a case-control design was assessed employing the NOS. A semi-ecological study, described by integrating ecological observations and individual-level data, can incorporate multiple control groups and is categorized as a case-control study. Hence, the NOS evaluated the semi-ecological study to discern its quality.

The score indicates that the quality of the included studies is moderate to high. Among the cohort studies, three studies scored 6 points, and two studies scored 7 points (Table 4). The case-control studies were scored 6 to 7 (Table 5), and the cross-sectional studies were scored 5 to 8 (Table 6).

**Table 4:** Quality assessment of cohort studies

First authors	Year	Country	Study type	Selection				Comparability	Outcome			Total score	Quality
				A	B	C	D	E	F	J	H		
Shapiro	2015	Canada	Cohort study	1	1	1	1	1	1	0	0	6	Moderate
Ashley-Martin	2018	Canada	Cohort study	1	1	1	1	1	1	0	0	6	Moderate
Xia	2018	China	Cohort study	1	1	1	1	1	1	0	1	7	High
Wang	2019	China	Cohort study	1	1	1	1	1	1	0	0	6	Moderate
Wang	2020	China	Cohort study	1	1	1	1	1	1	0	1	7	High

A: Representativeness of the exposed cohort (0/1 point); B: Selection of the nonexposed cohort (0/1 point); C: Ascertainment of exposure (0/1 point); D: Demonstration that outcome of interest was not present at the start of the study (0/1 point); E: Comparability of cohorts based on the design or analysis (0/1/2 point); F: Assessment of outcome (0/1 point); G: Was follow-up long enough for outcomes to occur (0/1 point); H: Adequacy of follow-up of cohorts (0/1 point)

**Table 5:** Quality assessment of case-control studies

First authors	Year	Country	Study type	Selection				Comparability	Outcome			Total score	Quality
				A	B	C	D	E	F	J	H		
Peng	2015	China	Retrospective case-control study nested in a cohort	1	1	0	1	1	1	1	0	6	Moderate
Marie	2018	French	Semi-ecological study	1	1	0	1	1	1	1	0	6	Moderate
Chen	2021	America	Case-control study	1	1	1	1	1	1	1	0	7	High
Jia	2021	China	Retrospective case-control study nested in a cohort	1	1	1	1	1	1	1	0	7	High

A: Is the case definition adequate? (0/1 point); B: Representativeness of the cases (0/1 point); C: Selection of Controls (0/1 point); D: Definition of Controls (0/1 point); E: Comparability of cases and controls based on the design or analysis (0/1/2 point); F: Ascertainment of exposure (0/1 point); G: Same method of ascertainment for cases and controls (0/1 point); H: Nonresponse rate (0/1 point)

**Table 6:** Quality assessment of cross-sectional studies

First authors	Year	Country	Study type	Item											Total score	Quality
				A	B	C	D	E	F	J	H	I	J	K		
Khan	2018	Bangladesh	Cross-sectional study	1	1	0	1	1	0	0	0	0	1	0	5	Moderate
Muñoz	2018	Chile	Cross-sectional study	1	0	0	1	1	0	1	1	0	1	0	6	Moderate
Zhang	2021	China	Cross-sectional study	1	1	1	1	1	0	1	1	0	1	0	8	High
Liang	2023	China	Cross-sectional study	1	1	1	1	1	0	1	1	0	1	0	8	High

A: Define the source of information (0/1 point); B: List inclusion and exclusion criteria for exposed and unexposed subjects (cases and controls) or refer to previous publications (0/1 point); C: Indicate time period used for identifying patients (0/1 point); D: Indicate whether or not subjects were consecutive if not population based (0/1 point); E: Indicate if evaluators of subjective components of study were masked to other aspects of the status of the participants (0/1 point); F: Describe any assessments undertaken for quality assurance purposes (0/1 point); G: Explain any patient exclusions from analysis (0/1 point); H: Describe how confounding was assessed and/or controlled (0/1 point); I: If applicable, explain how missing data were handled in the analysis (0/1 point); J: Summarize patient response rates and completeness of data collection (0/1 point); K: Clarify what follow-up, if any, was expected and the percentage of patients for which incomplete data or follow-up was obtained (0/1 point)

## Discussion

This meta-analysis aimed to summarize the main studies on the correlation between As and GDM incidence, revealing the relationship between As exposure and GDM. However, considering the heterogeneity of merged studies, a detailed explanation of the results is crucial. In addition, sensitivity analysis revealed that despite significant heterogeneity, the results were statistically significant and robust. Subgroup analyses indicated that heterogeneity could be attributed to the year of publication.

As an increasing number of young women are diagnosed with hyperglycemia or overt diabetes, people are becoming increasingly worried about GDM. It is caused by several risk factors, including obesity, previous GDM history, and familial history of type 2 diabetes [40]. The pathophysiology of GDM is multifactorial and involves both genetic predispositions and environmental triggers that contribute to its development. A key factor in the pathogenesis of GDM is insulin resistance [41], which is exacerbated during pregnancy due to hormonal changes, leading to inadequate insulin compensation and hyperglycemia.

Recent studies have highlighted the role of assisted reproductive technology (ART) as a significant variable in assessing GDM risk. Women who conceive through ART may have a predisposition to metabolic disorders, including GDM, due to underlying factors such as polycystic ovary syndrome (PCOS) and endometriosis, which are often associated with infertility treated by ART. Furthermore, the hormonal treatments involved in ART, such as ovulation induction, can alter glucose metabolism, increasing the risk of developing GDM. The influence of ART on GDM risk underscores the importance of considering the mode of conception in the assessment and management of GDM. For instance, a meta-analysis [42] stratified by mode of conception, disease location, and severity revealed a greater risk of GDM in women with endometriosis, especially those who conceived through ART, than in those conceived through natural conception. These findings suggest that ART may compound the risk of GDM in women already predisposed to pregnancy due to underlying reproductive disorders.

Moreover, demographic and clinical data play a crucial role in understanding the risk factors and mechanisms underlying GDM. Incorporating detailed demographic and clinical data, including prepregnancy weight, age, family history of diabetes, and previous GDM history, can provide insights into the individual risk profiles of pregnant women. A comprehensive approach that includes these variables, as detailed in recent research [43], can enhance our understanding of the multifaceted etiology of GDM and improve risk stratification and management strategies.

Building on this foundation, the variability of GDM diagnostic criteria poses certain challenges. The criteria proposed by the International Association of Diabetes and Pregnancy Study Groups (IADPSG) have been widely advocated, yet significant disagreements exist in the established diagnostic standards for GDM [44, 45]. These criteria are based on the 75-g oral glucose tolerance test (OGTT), which diagnoses GDM when fasting blood glucose is ≥ 5.1 mmol/L, 1-hour blood glucose is ≥ 10.0 mmol/L, or 2-hour blood glucose is ≥ 8.0 mmol/L. Some approved standards and guidelines exist, such as those from the World Health Organization (WHO) and the Australian Diabetes Association (ADIPS). Blood glucose thresholds differ at different times [46]. This disagreement highlights the various interpretations and implementations of GDM diagnosis in different regions. Throughout pregnancy, changes in glucose regulation are crucial for ensuring adequate nutrition for the fetus. Existing studies using high-insulin normoglycemic clamps have shown a 56% decrease in insulin sensitivity and a 30% increase in basal endogenous glucose production during the advanced stages of pregnancy. To counterbalance these changes, pancreatic β-cells enhance insulin secretion to maintain blood glucose stability [47]. Further studies suggested that any insulin resistance correlated with a normal pregnancy will rapidly reverse after delivery, indicating the regulatory role of placental factors in these physiological changes. A link between the environmental pollutant As and GDM has been established, as As can penetrate the placental barrier and affect glucose metabolism in both mothers and fetuses [2].

The universal correlation of As with numerous pathological conditions has attracted global concern, as As affects countries at all levels of development. Despite the severe health risks associated with As exposure, contact is often unintentional, primarily because of the consumption of contaminated water and food once inside the body. It induces metabolic disorders through multiple pathways and causes inflammatory responses, triggering oxidative stress and inflammation [48]. Moreover, As impairs glucose tolerance predominantly through the dysfunction of insulin-secreting β-cells rather than through an increase in peripheral insulin resistance [49]. The liver can use the methyl group in S-adenosylmethionine (SAM) to methylate inorganic As, forming monomethyl arsenic acid (MMA) and dimethyl arsenic acid (DMA), both of which are organic As with reduced toxicity [50]. Therefore, a higher efficiency of methylation results in lower toxicity due to increased conversion rates. Understanding the differences in toxicity between organic and inorganic As is crucial. Some inorganic As is metabolized into o-arsenate (As^3+^), a highly toxic metabolite linked to As poisoning. The metabolized As are primarily excreted through the kidneys. Some are excreted through the urine, and others are excreted through the bile. Strict mitigation measures are needed, as they can penetrate various biological barriers and affect multiple organs and systems.

Previous observational studies have suggested an indirect association between GDM and As exposure [17–20, 30, 31, 33]. Conversely, recent studies suggest that this relationship lacks statistical significance [22–25]. Given these different findings, we performed this meta-analysis of the latest evidence to explain the degree of connection between GDM and As exposure. The studies published between 2021 and 2023, cited by Chen et al. [22], had no statistically significant associations. They concluded that there was no significant association between total inorganic As and GDM (OR 0.77, 95% CI: 0.33 to 1.79). Similarly, Jia et al. [23] measured As levels in hair and found no substantial association between As and GDM (OR 0.91, 95% CI: 0.77 to 1.07). Furthermore, Zhang et al. [24] studied the combined impact of As and one-carbon metabolism (OCM) on GDM using urine. They found no significant association between total As and GDM (OR 0.90, 95% CI: 0.44 to 1.82). Another recent study in China by Liang et al. [25] used urine measurements and found no significant association between inorganic As and GDM (OR 0.66, 95% CI: 0.30 to 1.45). Considering the inconsistent results in the literature, this emphasizes the importance of ongoing investigations and rigorous analyses.

In the preliminary meta-analysis of As and GDM, nine studies involving 1,984 GDM patients were merged [51]. The final result (OR 1.56, 95% CI: 1.23 to 1.99) had significant heterogeneity (*I*^2^ = 64%). This can be caused by the use of different samples, including blood, urine, tap water, meconium, and toenails. Our comprehensive findings indicate that As increases the risk of GDM (OR 1.47, 95% CI: 1.11 to 1.95), with considerable heterogeneity (*I*^2^ = 76%). Although our results of this meta-analysis are consistent with the previous meta-analysis, the effect size has slightly decreased. Additionally, Egger’s test indicated potential publication bias, possibly due to selective reporting and publication preferences. Sensitivity analysis confirmed the robust results, while subgroup analysis explored the sources of heterogeneity. The national subgroups produced different results, possibly due to differences in As intake due to differences in the environment and dietary habits. Metabolic processes and genetic factors may further influence As intake. Subgroup analyses of thresholds revealed statistical significance only for quartile thresholds. The diagnostic criteria, study design, and sample type could not explain the source of heterogeneity. However, the year of publication may provide some explanation for the source of heterogeneity. Future studies could consider the impact of As on GDM by determining diagnostic criteria and dietary habits. While our current analysis of the studies is limited, it is still critical to conduct such studies in the future.

This meta-analysis has many strengths. First, combining data from 13 studies increased the sample size to 2575, improving the accuracy of our findings. Second, the included studies were of medium-to-high quality and were evaluated by the appropriate scales, ensuring highly reliable and credible data. Third, this study conducted a detailed analysis of the subgroups to explore the sources of heterogeneity.

However, this study has several limitations. First, due to differences in diagnostic criteria, test samples, and study designs, there was significant heterogeneity among the studies. Second, the study had publication bias, meaning that there was unpublished literature. Future efforts should integrate these neglected studies to evaluate the overall impact of As on GDM more comprehensively. Third, the use of multiple subgroups results in a smaller sample size, reducing the statistical power and complicating the clarification of effects. Therefore, the heterogeneity among the included studies was not fully explained. Nevertheless, based on existing studies, the current study represents an estimate of the association between As and GDM and provides additional information on different subgroups. However, additional large-scale studies are needed in the future to verify these results.

## Conclusions

This meta-analysis suggested that pregnant women with As carry a considerable risk of developing GDM. However, due to significant heterogeneity between studies and sample differences, careful interpretation is necessary. Selecting a single sample in the future is essential for drawing more accurate and reliable conclusions.

### Electronic supplementary material

Below is the link to the electronic supplementary material.

Supplementary Material 1: Search strategy for electronic databases.

Supplementary Material 2: Datasets.

Supplementary Material 3: PRISMA checklist.

## Data Availability

No datasets were generated or analysed during the current study.

## Abbreviations

GDM: Gestational Diabetes Mellitus
As: Arsenic
LOD: Limit of detection
WHO: World Health Organization
CDA-SOGC: Canadian Diabetes Association-Society of Obstetricians and Gynecologists of Canada
CNGOF: French National College of Obstetricians and Gynecologists
ADA: American Diabetes Association
MOH: Ministry of Health of China
SAM: S-adenosylmethionine
CAU-As: Creatinine-adjusted urinary arsenic
MMA: Monomethylarsonic acid
DMA: Dimethylarsinic acid AsB and Arsenobetaine
IADPSG: International Association of Diabetes and Pregnancy Study Groups
OGTT: Oral glucose tolerance test
