# Supplementary material for: Effect of arsenic on the risk of gestational diabetes mellitus: a systematic review and meta-analysis
Source: BMC Public Health. 2024 Apr 23;24:1131. doi: 10.1186/s12889-024-18596-6 (PMC11041030; doi:10.1186/s12889-024-18596-6)
Supplement: Supplementary file 3 — Supplementary Material 3: PRISMA checklist. [file 12889_2024_18596_MOESM3_ESM.docx]

**Supplementary Information**

Effect of arsenic on the risk of gestational diabetes mellitus: a systematic review and meta-analysis

Rui Wu^1^ , Min Duan^1^, Dongsheng Zong^2^*, and Zuojing Li^2^*

^1^School of Life Sciences and Biopharmaceuticals, Shenyang Pharmaceutical University, Shenyang, China

^2^School of Medical Devices, Shenyang Pharmaceutical University, Shenyang, China

*****Correspondence: zuojing1006@hotmail.com;15040274832@163.com.

# Search strategies

| **Database** | **Search strategy** | **Results** |
| --- | --- | --- |
| **Pubmed** | **(("Diabetes, Gestational"[Mesh] OR Diabetes, Pregnancy-Induced[Title/Abstract] OR Diabetes, Pregnancy Induced[Title/Abstract] OR Pregnancy-Induced Diabetes[Title/Abstract] OR Gestational Diabetes[Title/Abstract] OR Diabetes Mellitus, Gestational[Title/Abstract] OR Gestational Diabetes Mellitus[Title/Abstract])) AND (("Arsenic"[Mesh] OR Arsenic-75[Title/Abstract] OR Arsenic 75[Title/Abstract]))** | **19** |
| **EMBASE** | **('pregnancy diabetes mellitus'/exp OR ‘Diabetes, Pregnancy-Induced’:ti,ab OR ‘Diabetes, Pregnancy Induced’:ti,ab OR ‘Pregnancy-Induced Diabetes’:ti,ab OR ‘Gestational Diabetes’:ti,ab OR ‘Diabetes Mellitus, Gestational’:ti,ab OR ‘Gestational Diabetes Mellitus’:ti,ab) AND ('Arsenic'/exp OR ‘Arsenic-75’:ti,ab OR ‘Arsenic 75’:ti,ab)** | **60** |
| **Scopus** | **( TITLE-ABS-KEY ( ( "pregnancy diabetes mellitus" OR "Diabetes, Pregnancy-Induced" OR "Diabetes, Pregnancy Induced" OR "Pregnancy-Induced Diabetes" OR "Gestational Diabetes" OR "Diabetes Mellitus, Gestational" OR "Gestational Diabetes Mellitus" ) ) ) AND ( TITLE-ABS-KEY ( ( "Arsenic" OR "Arsenic-75" OR "Arsenic 75" ) ) )** | **56** |

*** All searches were carried out on September 8, 2023.**
